# *In Vivo* Photoadduction of Anesthetic Ligands in Mouse Brain Markedly Extends Sedation and Hypnosis

**DOI:** 10.1523/JNEUROSCI.1884-22.2023

**Published:** 2023-03-29

**Authors:** Andrew R. McKinstry-Wu, Andrzej Z. Wasilczuk, William P. Dailey, Roderic G. Eckenhoff, Max B. Kelz

**Affiliations:** ^1^Department of Anesthesiology and Critical Care, University of Pennsylvania Perelman School of Medicine, Philadelphia, Philadelphia 19104; ^2^Department of Bioengineering, University of Pennsylvania, Philadelphia, Philadelphia 19104; ^3^Department of Chemistry, University of Pennsylvania School of Arts and Sciences, Philadelphia, Pennsylvania 19104; ^4^Mahoney Institute for Neurosciences, University of Pennsylvania, Perelman School of Medicine, Philadelphia, Philadelphia 19104; ^5^Neuroscience of Unconsciousness and Reanimation Research Alliance, University of Pennsylvania, Perelman School of Medicine, Philadelphia, Philadelphia 19104

**Keywords:** anesthesia, hypnosis, locus coeruleus, neuronal mechanisms, photo affinity ligand, sedation

## Abstract

Photoaffinity ligands are best known as tools used to identify the specific binding sites of drugs to their molecular targets. However, photoaffinity ligands have the potential to further define critical neuroanatomic targets of drug action. In the brains of WT male mice, we demonstrate the feasibility of using photoaffinity ligands *in vivo* to prolong anesthesia via targeted yet spatially restricted photoadduction of azi-*m*-propofol (aziPm), a photoreactive analog of the general anesthetic propofol. Systemic administration of aziPm with bilateral near-ultraviolet photoadduction in the rostral pons, at the border of the parabrachial nucleus and locus coeruleus, produced a 20-fold increase in the duration of sedative and hypnotic effects compared with control mice without UV illumination. Photoadduction that missed the parabrachial-coerulean complex also failed to extend the sedative or hypnotic actions of aziPm and was indistinguishable from nonadducted controls. Paralleling the prolonged behavioral and EEG consequences of on target *in vivo* photoadduction, we conducted electrophysiologic recordings in rostral pontine brain slices. Using neurons within the locus coeruleus to further highlight the cellular consequences of irreversible aziPm binding, we demonstrate transient slowing of spontaneous action potentials with a brief bath application of aziPm that becomes irreversible on photoadduction. Together, these findings suggest that photochemistry-based strategies are a viable new approach for probing CNS physiology and pathophysiology.

**SIGNIFICANCE STATEMENT** Photoaffinity ligands are drugs capable of light-induced irreversible binding, which have unexploited potential to identify the neuroanatomic sites of drug action. We systemically administer a centrally acting anesthetic photoaffinity ligand in mice, conduct localized photoillumination within the brain to covalently adduct the drug at its *in vivo* sites of action, and successfully enrich irreversible drug binding within a restricted 250 µm radius. When photoadduction encompassed the pontine parabrachial-coerulean complex, anesthetic sedation and hypnosis was prolonged 20-fold, thus illustrating the power of *in vivo* photochemistry to help unravel neuronal mechanisms of drug action.

## Introduction

Photochemistry offers an opportunity to take advantage of therapeutic polypharmacology while minimizing potential adverse effects by limiting drug effect to circumscribed regions. Oncology has used photochemistry in precisely this way: though chemotherapies often mechanistically target nonspecific processes, spatially restricting these drugs maximizes desirable therapeutic effects while limiting adverse ones (Pasparakis et al., 2014). While photo-activated chemotherapies have primarily been deployed *in vitro* to date, photo-activated opioids have been used in *in vivo* models to produce local analgesia while avoiding undesirable systemic effects (López-Cano et al., 2021). Conversely, photoaffinity ligand derivatives of general anesthetics, antipsychotics, and antidepressants have been used successfully *in vitro* to investigate drug–receptor interactions (Savechenkov et al., 2012, 2017; Stein et al., 2012; Budelier et al., 2017; Chen et al., 2019; Sugasawa et al., 2019, 2020), but have not been deployed *in vivo* in mammals to extend or enhance drug effects while limiting off-target effects (Zawarynski et al., 1998; Chiara et al., 2003; Henry et al., 2006; G. D. Li et al., 2006; Sanghvi et al., 2008; McKinstry-Wu et al., 2019).

General anesthetics provide an ideal model for *in vivo* photoadduction of a CNS therapeutic. Anesthetics and their corresponding photoaffinity ligands produce unambiguous, readily observable behavioral effects as well as specific changes at the single-neuron level. They are delivered systemically to produce loss of consciousness (hypnosis) and have therapeutic and off-target effects that demonstrate anatomic selectivity (Devor and Zalkind, 2001; Lazarenko et al., 2010; Zhou et al., 2013). We hypothesized that localized photochemical adduction would permit neuroanatomical exploration of the drug's desirable effects without the confounds of direct drug microinjections that potentially generate supratherapeutic concentrations. Consequently, we hypothesized that anesthetic photoaffinity ligands would offer a quintessential methodology for studying the effects of regional photoadduction on neuronal activity and behavior.

Propofol is a general anesthetic member of the alkylphenol class of anesthetics and is the most commonly used clinical anesthetic. The desired hypnotic actions of propofol and other alkylphenols are accompanied by potentially lethal effects, including respiratory and cardiovascular depression, which limit their therapeutic indices. While the molecular targets mediating propofol-induced hypnosis and adverse effects may not be fully dissociable (Jurd et al., 2003; Zeller et al., 2005), the neuronal populations likely are. Several photolabel analogs of propofol produce prolonged hypnosis with whole-organism photolabeling in tadpoles (Hall et al., 2010; Stewart et al., 2011; Weiser et al., 2013; Yip et al., 2013; Chen et al., 2014), prompting the question of potential efficacy of spatially restricted drug action.

The rostral pons is home to multiple arousal centers known to interact with anesthetics. The locus coeruleus (LC), a pontine adrenergic center with widespread cortical and subcortical connections, has a well-established and significant role in mediating transitions from hypnosis to waking (Aston-Jones and Bloom, 1981; Carter et al., 2010; Vazey and Aston-Jones, 2014; Nir et al., 2019). As a known positive allosteric modulator of GABA_A_ receptors, propofol inhibits activity of neurons in the LC, both directly and indirectly (Chen et al., 1999; Y. Zhang et al., 2015). Neighboring parabrachial nuclei also regulate arousal from sleep and from anesthetized states (Fuller et al., 2011; Fischer et al., 2016; Muindi et al., 2016; Qiu et al., 2016; Luo et al., 2018; Wang et al., 2019). Given propofol's known actions on pontine arousal-promoting neurons, we hypothesized that localized fiber-optic near-ultraviolet (UV) illumination of a propofol-based photoaffinity ligand within the rostral pons would cause irreversible covalent binding at important *in vivo* sites of action and consequently prolong the anesthetic state. We demonstrate that covalently trapping the anesthetic photoaffinity ligand, azi-*m*-propofol (aziPm) using UV photoillumination at the interface of the LC and parabrachial nucleus prolongs the duration of anesthetic hypnosis 20-fold. Electrophysiologic recordings in slice confirm a cellular corollary wherein photoadduction of aziPm irreversibly prolongs the inhibitory effects. Cumulatively, our data demonstrate that adduction of photoaffinity ligands locally prolongs drug effects at the neuronal level *in vitro* and is sufficient *in vivo* to prolong desired drug effects behaviorally and electroencephalographically.

## Materials and Methods

### Photoligand synthesis and formulation

aziPm was synthesized to >98% purity per previously published methods (Hall et al., 2010) and shipped to AmBios Labs for tritiation to produce ^3^H-aziPm. ^3^H-aziPm was confirmed to be 98% pure by HPLC. All stock aziPm, ^3^H-aziPm, and propofol were suspended in a vehicle of 20% β-cyclodextrin in dH_2_O.

### Animals

All studies were approved by the Institutional Animal Care and Use Committee at the University of Pennsylvania and were conducted in accordance with National Institutes of Health guidelines. Male WT C57BL/6 mice (The Jackson Laboratory) 8-12 weeks of age were used for studies of UV light penetration through the brain *in vitro* (*n* = 2), central venous implant and aziPm dose–response studies (*n* = 15), chronic EEG implantation and *in vivo* photoadduction (*n* = 30), tritiated aziPm *in vivo* photoadduction (*n* = 3), and whole-cell electrophysiologic recordings (*n* = 12). Mice were habituated to a reverse 12:12 light-dark cycle for at least 2 weeks before studies such that all testing occurred during their active period.

### Propofol and aziPm dose–response

In order to retest the same animal over time with randomized drug dosing, a subset of mice underwent jugular cannulation per published protocols (Shortal et al., 2018). Mice implanted with central lines were randomized to receive varying doses of propofol, ranging from 5 to 30 mg/kg and to varying doses of aziPm, ranging from 20 to 60 mg/kg. Mice were briefly restrained during drug injection, then immediately removed and tested for righting reflex, while maintaining temperature using a heating pad. Animals that failed to right themselves within 5 s of being placed supine were considered to have lost their righting reflex. Population dose–response curves for propofol and aziPm were generated using MATLAB by fitting a Hill equation and minimizing the sum of squared error between the dose–response estimate and observed data (McKinstry-Wu et al., 2019).

### UV power decay through brain tissue

Unfixed brain tissue from two mice was sliced with a Leica Vibratome (VT1200) at varying thickness ranging from 200 to 1000 μm in 200 μm increments. This tissue was used to establish the relationship of laser power through the brain as a function of tissue depth. Unfixed tissue was rapidly placed atop the slide power meter. Laser power was repeatedly measured through the brain slices of varying thickness with a microscope slide power meter sensor head (S170C and PM100D, Thorlabs). Between readings, laser power was confirmed to be stable and unchanging in the absence of any unfixed brain tissue (0 μm thickness).

### *In vivo* ^3^H-aziPm photoadduction and autoradiography

Mice (*n* = 3) were implanted with bilateral guide cannulae and given at least 1 week to recover before further experimentation. Thereafter, mice were briefly restrained while a 30 gauge tail vein intravenous line was placed. Dual optical fibers were secured through implanted guide cannula targeting coordinates of 3.00 mm posterior, 2.00 mm ventral, and ±0.8 mm lateral with respect to bregma. As UV power penetrating through tissue ultimately controls photoadduction, to determine the extent of *in vivo* photoadduction, mice received 1 mg/kg ^3^H-aziPm via tail vein catheter infused over 5 min with 15 mW of 375 nm light delivered continuously during the final minute of infusion. After 2 h of recovery to permit elimination of nonphotoadducted ^3^H-aziPm, mice were killed and transcardially perfused PBS followed by 4% PFA. Brains were subsequently postfixed for 24 h in 4% PFA before being cut into 50 μm sections. Sections were further washed in PBS 5 times to remove any remaining unbound drug before slide mounting and drying. Slide-mounted, dried brain sections containing photoadducted by ^3^H-aziPm and tritium standards (American Radiolabeled Chemicals) were placed on a tritium phosphor screen (GE Healthcare Life Sciences) in an autoradiography cassette (Fisher Scientific) and exposed for 7 d before screen development. The exposed phosphor screen was developed using the Personal Molecular Imager FX system (Bio-Rad).

### Quantification of *in vivo* ^3^H-aziPm photoadduction

Digital autoradiographic images were analyzed in MATLAB (2022a, The MathWorks) using custom code incorporating the image processing toolbox. Mean radiograph pixel intensities were taken for each standard and used to create a standard curve relating radiograph pixel intensity and ^3^H-aziPm concentration. An ROI was then defined for each coronal section having pixel intensities greater than background tritiated signal intensity. Signal intensity was then converted to estimated ^3^H-aziPm concentration using the tritium standard curve for each ROI. The intensity and distribution of *in vivo* photoadduction were computed assuming a spherical model of photoadduction spread. ^3^H-aziPm concentration was expressed as a function of radial distance was calculated assuming that the maximal signal intensity was at the epicenter of the sphere (see Fig. 3*C*). Signal intensity as a function of polar distance was fit to a two-parameter Hill equation, and 95% CIs were estimated from the Jacobian of the nonlinear regression model.

### EEG, EMG, and cannula implantation

EEG headpieces and EMG leads were constructed and implanted as previously described (Wasilczuk et al., 2016), with the following modifications: implanted headstages contained bilateral 2 × 5 rows of pins, with 16 total EEG leads arranged at 1.3 mm intervals from 2.6 mm anterior to bregma to 2.6 mm posterior to bregma and two cervical EMG leads implanted on each side to fashion a bilateral array. EEG leads were located 0.7 and 2.0 mm lateral to midline, with leads in each hemisphere. An anchor screw was implanted at 3 mm posterior to bregma and 3.5 mm left-lateral.

With the mouse still in the stereotaxic frame, future fiber-optic access was created bilaterally by drilling 0.7-mm-diameter holes at 6.5 mm posterior to bregma ± 0.8 mm lateral. A paired 23 gauge guide cannula (Plastics One) extending 2 mm below the attached pedestal was implanted through these holes at a 16 degree cephalad angle with internal dummy cannula extending an additional 1.2 mm beyond the guide cannula. Guide cannulae, pedestal, and EEG/EMG headpiece were secured with dental cement. Mice recovered for a minimum of 1 week following surgery before any experimentation was conducted.

### Tape removal test

Beginning 1 week after surgery, mice underwent daily training to remove a 5-mm-diameter circular adhesive sticker placed on their noses. Timing of both attempted (any purposeful movement of a paw toward the nose) and successful tape removal was recorded. Mice were considered trained when they removed the adhesive sticker in <10 s (Wasilczuk et al., 2018). The duration of the anesthetic state was quantified by recording the time for sticker removal in fully trained mice from end of the anesthetic drug infusion to attempted removal and successful removal was recorded. One of 30 mice failed to reach the baseline goal of removing the sticker in <10 s and was consequently excluded from all further analyses.

### aziPm infusion and intracranial UV light exposure

Mice received intravenous aziPm with intracranial photoadduction as previously described (McKinstry-Wu and Kelz, 2018). After performing a baseline sticker removal test, mice were restrained, attached to a recording headstage, and a 30 gauge tail vein intravenous line was placed and secured. Dual optical fibers (200 μm core, Thorlabs) extending 1.2 mm beyond the terminals of the guide cannulae were inserted through the cannulae. After 10 min of baseline EEG recording (see below), 15 mW of 375 nm laser light (200 mW 375 nm fiber-coupled laser, Power Technology) was delivered via the attached optical fibers and another 10 min baseline EEG recorded; 100 mg/kg aziPm was administered intravenously over 5 min via syringe pump (Harvard 11 Elite, Harvard Apparatus). In a subset of animals (*n* = 19), 15 mW 375 nm light was administered via the optical fibers during the last minute of drug infusion, while the remainder (*n* = 11) did not receive 375 nm photoillumination. Of the mice receiving photoadduction, 3 were excluded from analyses: 1 because of tail vein intravenous infiltration, 1 for death concurrent with laser illumination, and 1 because of inability to localize implanted cannulae. One of the mice not receiving photoillumination was excluded from further analyses because of inability to perform tape removal test in under 10 s before drug administration. Mice were removed from the restraint, an adhesive sticker placed on their snout, as described above, and placed in a cage warmed by a heat lamp and under video recording. Electrophysiological recording continued for at least 20 min after recovery, defined as when the mice successfully removed the adhesive from their nose. Fiber power output was confirmed between experiments using a digital optical power meter with a fiber photodiode power sensor attachment (PM100D and S150C).

### Cannula localization

The day following aziPm exposure, to mark the location of the optical fiber delivering UV light, dummy cannulas extending 1.2 mm beyond the terminals of implanted guide cannulas were coated with DiI, a lipophilic membrane stain (V22885, Fisher Scientific), and inserted into the implanted animal's guide cannulae. Mice were killed and transcardially perfused with PBS, then 4% PFA. Brains were additionally postfixed for 24 h in 4% PFA before sectioning, slide mounting, and imaging. Cannula dummy tip placement was anatomically localized according to a stereotaxic brain atlas (Paxinos and Franklin, 2008) by three blinded scorers using the images based on DiI staining, where the most dorsal aspect of DiI staining was considered the location of the epicenter of the UV light source.

### Estimation of *in vivo* photoadduction

Based on the 50% decrement of photoadduction by 231 µm seen in the sections with ^3^H-aziPm (see Fig. 3), the biologically relevant concentrations of photolabeled aziPm were demarcated using a sphere with a conservatively larger 250 µm radius placed on the most ventral DiI-stained section of brain, which was considered to denote the epicenter of the UV light source. For categorization purposes, if the volume of the estimated adduction spheres in a given subject crossed the superior-lateral border of the LC with the parabrachial nuclei (between bregma −5.34 and bregma −5.68) bilaterally, that subject was considered to have photoadduction at the parabrachial-coerulean complex.

### EEG/EMG acquisition and analysis

EEG and EMG were recorded using Intan headstages (Intan Technologies) through an acquisition box constructed according to Open-Ephys specifications (www.open-ephys.org) (Siegle et al., 2017). All signals were recorded using the freely available open source open-ephys GUI acquisition software (www.open-ephys.org) and analyzed in MATLAB (2022a, The MathWorks) using custom code using the signal processing and statistics toolboxes. EEG preprocessing was conducted as previously described (Wasilczuk et al., 2021), except that bandpass filters with cutoffs of 0.5 and 100 Hz were used. After mean re-referencing, a single EEG lead overlying the left frontal association area (bregma 2.6 mm, 0.7 mm left lateral) was analyzed. Artifact-free 10 s nonoverlapping segments of EEG from each individual mouse were taken from 10 min periods before drug administration, 5 min after the beginning of drug infusion, and 45 min after the beginning of drug infusion (8-60 segments per period by individual.) Tapered spectral estimates (5 tapers) for each individual at the described periods were obtained using previously published code (Hudson et al., 2014), mean spectra by group calculated and error estimated with bootstrapping (100 bootstraps). Spectral slopes for all spectra, as described (Lendner et al., 2020), were calculated using the *polyfit* function in MATLAB for each individual nonoverlapping window. Nuchal EMG was high-pass filtered with a cutoff of 70 Hz, and 10 s, nonoverlapping windows were used to compute EMG basal tone throughout the duration of the recorded experiment. Heart rate was measured using the interbeat R-R interval, which was captured during the first 10 min following drug infusion by thresholding the EMG to detect peaks in the QRS complex. Respiratory rate was estimated from the phasic variability in QRS peaks (Charlton et al., 2018). QRS peaks were subjected to cubic spline interpolation. The resulting signal was zero-phase filtered, and interpeak intervals were computed estimates for respiratory rate.

### LC slice preparation and electrophysiology

Eight- to 12-week-old mice were killed by cervical dislocation followed by decapitation, and rapid brain harvest. The brain was placed in ice-cold preoxygenated sucrose solution consisting of 248 mm sucrose, 2.5 mm KCl, 1.25 mm NaH_2_PO_4_, 2.0 mm MgSO_4_, 2.5 mm CaCl_2_, 10 mm dextrose, and 26 mm NaHCO_3_; 200-μm-thick coronal sections were cut through the pons using a VT1200 Leica microslicer (Leica Microsystems) while the brain was continuously oxygenated in the ice-cold sucrose solution. Sections were transferred to a holding chamber containing aCSF continuously bubbled with 95% O_2_/5% CO_2_. The composition of the aCSF was 124 mm NaCl, 2.5 mm KCl, 1.25 mm NaH_2_PO_4_, 2.0 mm MgSO_4_, 2.5 mm CaCl_2_, 10 mm dextrose, and 26 mm NaHCO_3_. Slices were maintained at 37°C for 1 h, then kept at room temperature until recording.

Whole-cell voltage-clamp and current-clamp techniques were conducted as previously described (Moore et al., 2012). During recording, slices were maintained in a recording chamber continuously perfused with oxygenated 32°C aCSF at a rate of 2 ml/min. The LC was localized via comparison with the mouse stereotaxic atlas (Paxinos and Franklin, 2008).

After a cell was visualized using an infrared filter and differential interference contrast, a gigaohm seal was made with a recording electrode and then the membrane was ruptured to obtain a whole-cell recording. Electrodes had a resistance of 5-8 mΩ when filled with an intracellular solution of 130 mm K-gluconate, 5 mm NaCl, 10 mm phosphocreatine disodium salt, 1 mm MgCl_2_, 10 mm HEPES, 0.02 mm EGTA, 0.5 mm Na_2_GTP, 2 mm MgATP, and 0.1% biocytin (pH 7.3, 280-290 mOsm).

In current-clamp mode, cells were characterized using an Multiclamp 700 amplifier, a Digidata 1320 A/D converter, and pClamp 10 software (Molecular Devices). Electrophysiologic characterization of putative LC neurons consisted of determining a given cell's current–voltage (*I–V*) relationship, spike-frequency adaptation, and parameters of delayed excitation (X. Zhang et al., 2010). Briefly, the *I–V* relationship was examined by injecting a series of pulse hyperpolarizing and depolarizing currents, starting from −0.16 nA with increasing increments of 0.02 nA. Spike frequency adaptation was assessed through injection of a series of depolarizing currents and examining peak firing frequency, steady-state frequency, and the ratio of the two. Delayed excitation, the time delay between the end of current injection and the appearance of the first action potential (AP), was assessed from 0.01 to −0.11 nA in 0.02 nA steps. Spike frequency adaptation was assessed with applied constant depolarizing currents from 0 to 0.2 nA in 0.04 nA increments. Based on known properties of LC neurons (Williams et al., 1988; X. Zhang et al., 2010), we only proceeded with cells having the characteristic nonlinear *I–V* relationship, a delayed production of AP following hyperpolarization, and showing an increasing interval over time between APs with constantly applied depolarizing current.

Cells were continuously recorded in current-clamp mode. After obtaining baseline characteristics for at least 10 min, aziPm dissolved in aCSF to a final concentration of 10 μm was bath-applied over each slice for 3 min, followed by a 20 min washout. This low dose was chosen because of its ability to elicit reversible effects. After the washout, a second 3 min bath application of 10 μm aziPm in aCSF was applied. One minute into the second aziPm exposure, 375 nm laser light (Power Technology) at 60 mW/mm^2^ illuminated the slice via microscope optics for 30 s. A control group underwent sham laser (no illumination) for 30 s. The second drug administration was followed by a washout period of up to 2 h or for as long as the patch could be held. Based on *post hoc* analysis of electrode placement, along with immunohistochemistry visualizing biocytin and anti-TH antibody deposition, only recordings from adrenergic cells were included for analysis. Firing rate for each neuron was normalized to individual average baseline rate for comparisons across neurons.

### *Ex vivo* immunofluorescent staining/visualization

Following *ex vivo* studies, the 200 μm brain sections were fixed for 30 min in 4% PFA in PBS. Before staining, residual PFA was rinsed from the sections with three PBS washes. Sections were then permeabilized and blocked for 1 h at room temperature with 5% normal goat serum in PBS containing 0.4% Triton. Sections were incubated overnight at room temperature in blocking solution with AlexaFluor-633-conjugated streptavidin (1:1000, Fisher Scientific) and rabbit anti-TH (1:500, Millipore). Unbound antibody was washed before a 4 h incubation with a goat anti-rabbit secondary antibody conjugated to an Alexa-488 fluorescent dye (1:200, Fisher Scientific) to detect the TH antibody. Sections were mounted and coverslipped for visualization with a Leica TCS SP5 confocal microscope.

### Statistical analysis

Analyses were performed using custom-written code in MATLAB using the Statistics and Machine Learning or using Prism 9 (GraphPad). Statistical comparisons of groups were performed using unpaired two-tailed *t* tests, two-way ANOVA, or the nonparametric Kruskal–Wallis test. *p* < 0.05 was considered statistically significant for all comparisons. Non-normalized data are reported as medians with interquartile ranges shown in parentheses.

## Results

### Photoadduction of aziPm at the parabrachial-coerulean complex profoundly extends the duration of hypnosis

**Figure 1.**
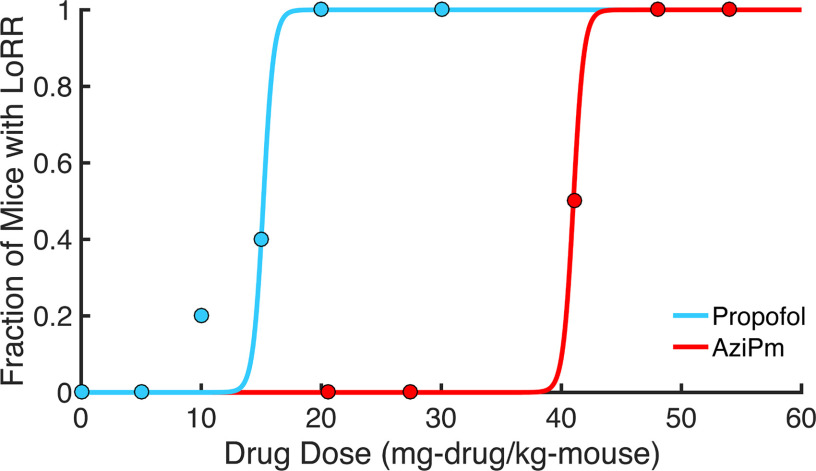
Dose–response curves for intravenous administration of propofol and aziPm. Dose–response curves were created for propofol (blue) and aziPm (red). Mice implanted with central venous catheters were subjected to randomly ordered doses of propofol (*n* = 5-7 per dose) or aziPm (*n* = 4-6 per dose), and loss of righting reflex was measured immediately after injection completion. The molar potency of aziPm (MW 244) was determined to be 0.51 times that of propofol (MW 178) based on the respective EC_50_ of each drug (EC_50propofol_ = 15.3 mg/kg, EC_50aziPm_ = 41.2 mg/kg). EC_50_ half maximal effective concentration MW, molecular weight.

**Figure 2.**
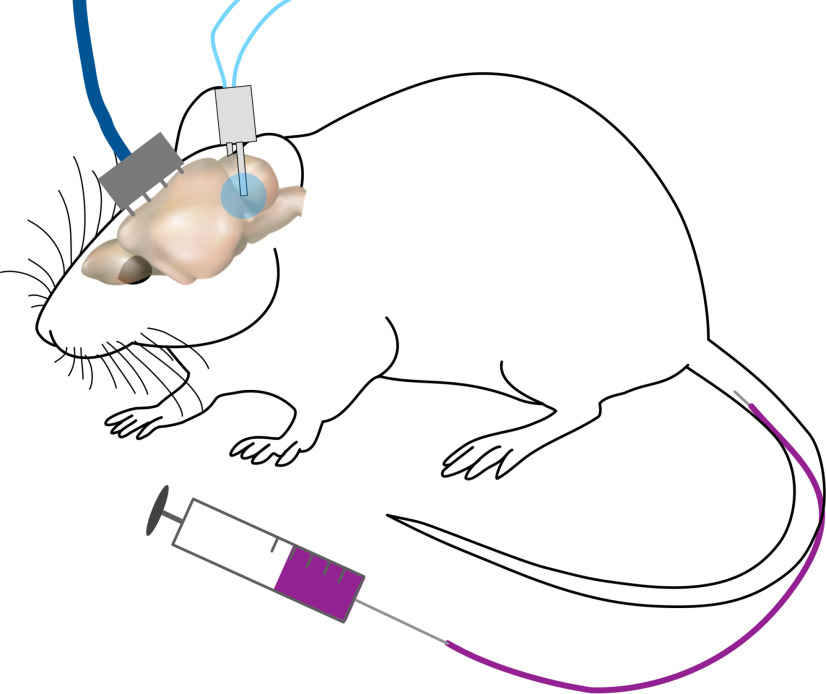
Experimental setup for *in vivo* photoadduction of aziPm or ^3^H-aziPm. Drug delivery was through tail vein (schematized in purple), and UV light photoillumination to locally adduct the drug was delivered through fiber-optic wires (schematized in blue). EEG and EMG were simultaneously recorded throughout experimentation.

**Figure 3.**
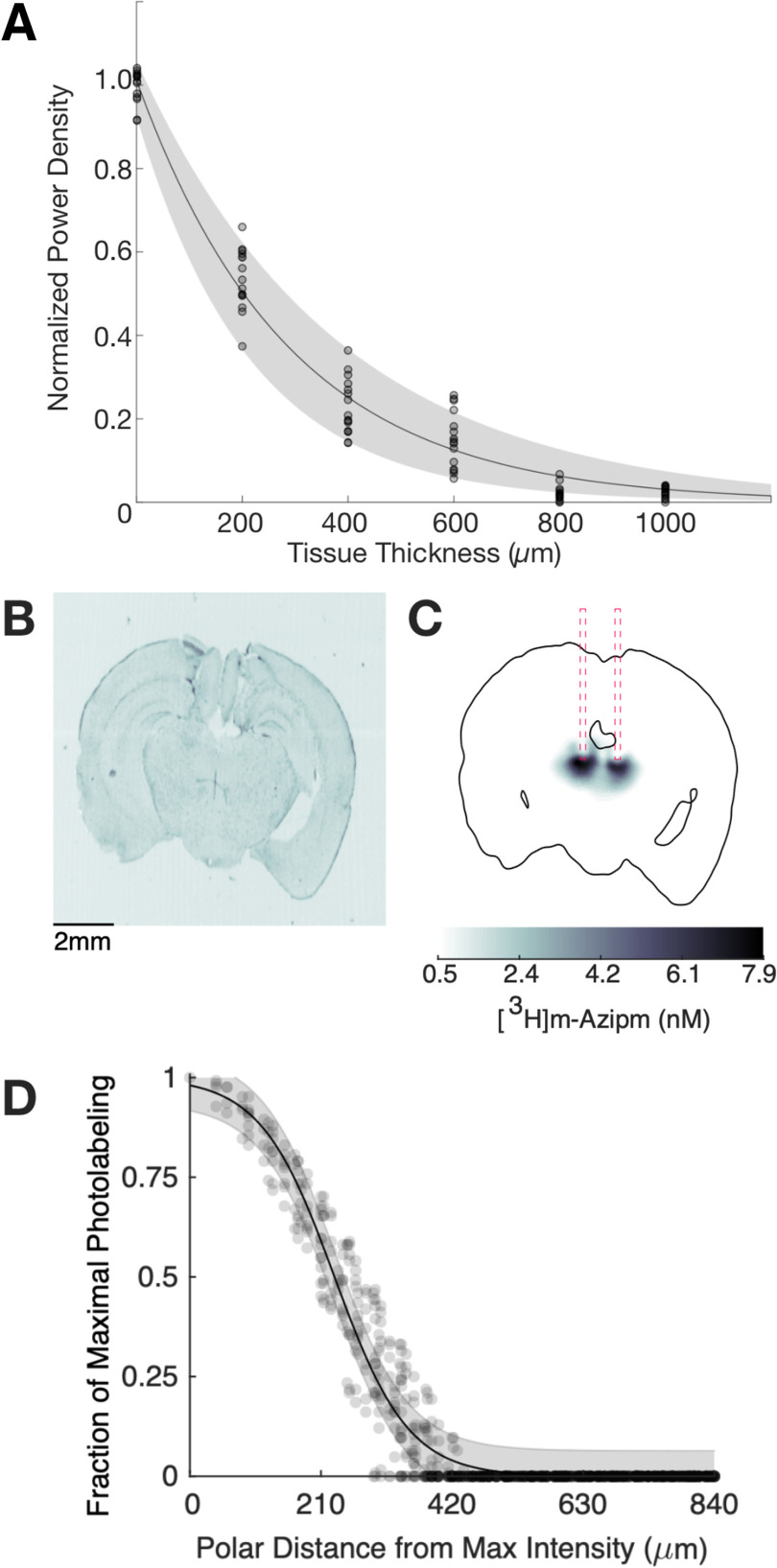
*In vivo* photoadduction produces neurophysiologically relevant concentrations of ^3^H-aziPm in a circumscribed region. ***A***, Laser power was plotted as a function of brain slice thicknesses in *ex vivo* experiments (16 measurements per thickness from 2 mice). Data were fit to an exponential decay function (*R*^2^ = 0.97). ***B***, Brightfield image and (***C***) corresponding tritium radiograph of a representative brain section from a photoadducted ^3^H-aziPm mouse demonstrate physiologically relevant levels of adduction immediately adjacent to the implanted optical fibers with a rapid decrease in [^3^H-aziPm] within a few hundred microns. ***D***, Spatial quantification of [^3^H-aziPm] was fit with the Hill equation (*R*^2^ = 0.97). Data are mean ± 95% CI. Gray circles represent discrete measurements of [^3^H-aziPm] taken across 146 50-µm coronal sections from 3 mice.

The hypnotic potency of intravenously administered aziPm was determined for WT C57BL6/J mice and found to be roughly half that of its parent compound, propofol (Fig. 1, EC_50_ for loss of righting 15.3 mg/kg for propofol vs 41.2 mg/kg for aziPm). To ultimately determine the spatial extent of adduction of anesthetic photoaffinity ligand in our mouse model (Fig. 2), we first estimated near-UV light penetration through brain. *Ex vivo* experiments measured laser power decay as a function of unfixed brain tissue thickness and found the 50% decrease in power occurred at 203 µm (Fig. 3*A*). To determine the extent of photoadduction *in vivo* in mice, 1 mg/kg of tritiated aziPm (^3^H-aziPm) was administered systemically through an intravenous tail vein injection, and brain parenchyma was illuminated locally using 375 nm laser light delivered fiberoptically via a chronically implanted guide cannula (Fig. 2). Mice were killed 2 h following a 5 min infusion of ^3^H-aziPm that coterminated with 1 min of UV photoillumination. This strategy allowed sufficient time for the drug to reach the brain, be locally photoadducted, and enabled nonphotoadducted ^3^H-aziPm to be eliminated. Radiographs revealed (Fig. 3*B*,*C*) that tissue concentration following photoadduction rapidly dropped with increasing polar distance from the fiber center. ^3^H-aziPm levels fell below 50% of maximal binding concentration by 231 µm (Fig. 3*C*). This steep spatial gradient demonstrates that localized photoadduction can be reliably used to target specific CNS structures.

As a behavioral marker of recovery from hypnosis, we measured the coordinated response to an inherent mildly aversive stimulus: removal of an adhesive sticker placed on the mouse's snout (Wasilczuk et al., 2018). The tape removal test is advantageous over other similar markers of recovery in that it can be conducted with mice in a prone position. Unlike the return of righting reflex that requires mice to be placed on their back, the tape removal test does not jeopardize the integrity of an indwelling fiber-optic cable. A trained, awake mouse will remove the adhesive sticker from its snout in <10 s.

**Figure 4.**
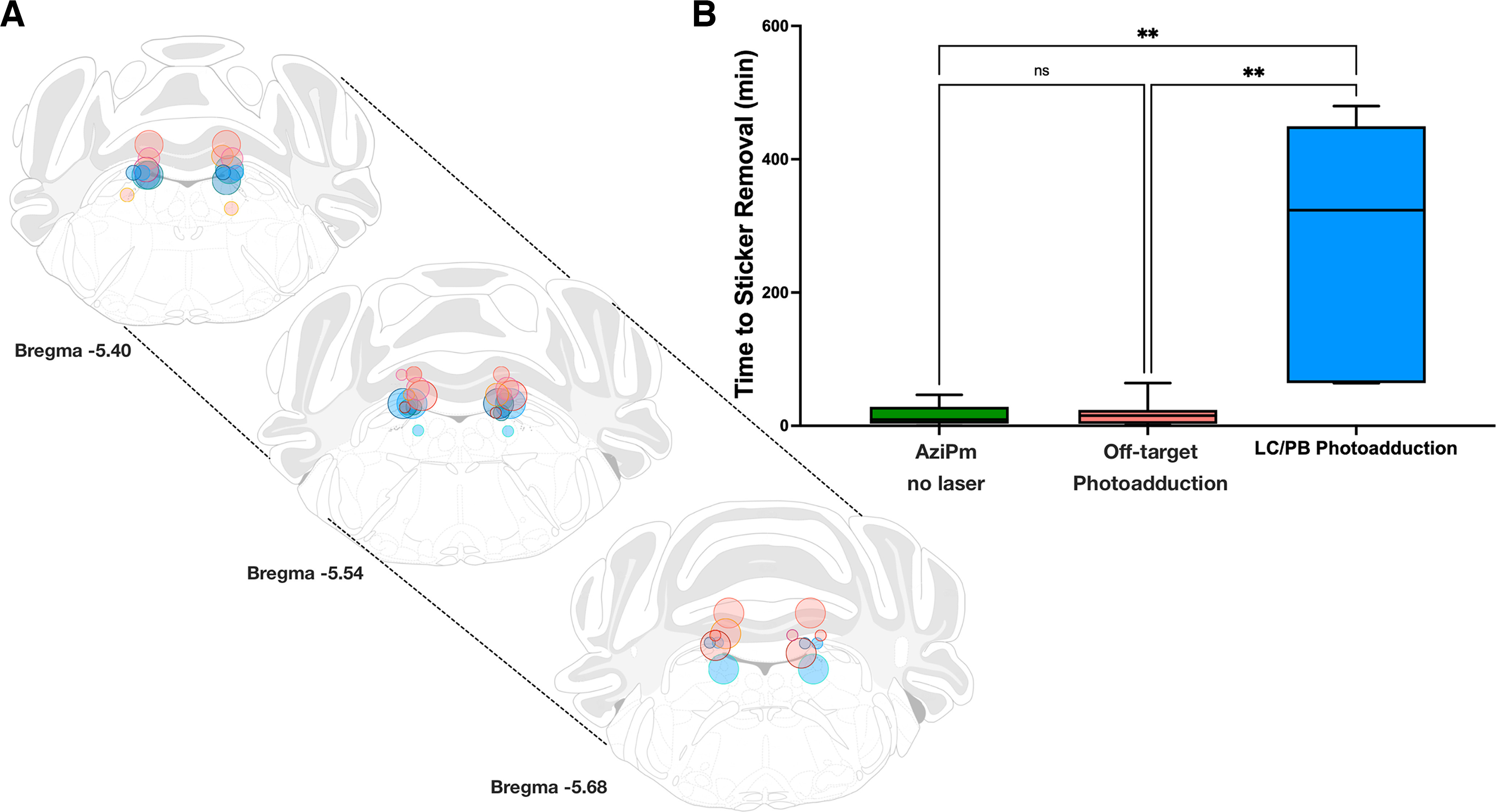
Photoadduction of aziPm at the parabrachial-coerulean junction profoundly extends the hypnotic state. ***A***, *Post hoc* localization of predicted photoadduction spheres within 250 µm of the parabrachial-coerulean complex (LC/PB group) are depicted over three rostral-caudal sections using blue face circles on each coronal brain slice (*n* = 5 mice). Red circles represent sites >250 µm from the parabrachial-coerulean junction (off-target photoadduction group, *n* = 11 mice). Individual mice are shown with unique circle edge colors. Three photoadducted mice were far off-target (outside of bregma −5.40 mm to bregma −5.68 mm) and consequently not displayed. ***B***, UV laser-induced photoadduction of aziPm encompassing the parabrachial-coerulean complex (LC/PB group in blue) markedly prolongs the duration of hypnosis as determined by adhesive tape removal testing compared with both off-target aziPm photoadduction (red group) and aziPm without any UV illumination (green group). **p < 0.01.

Mice that received 20 mg/kg/min of aziPm for 5 min (see Materials and Methods) without photoadduction reached the recovery endpoint, removal of tape, with a median (interquartile range) of 9.54 min (3.15-28.67 min). Delayed recovery was defined whenever mice took longer than 99% of animals receiving aziPm without UV light. Based on distributions shown in Figure 4, this corresponded to sticker removal times exceeding 46.5 min. Delayed recovery occurred in a subset (*n* = 6) of all the animals receiving 375 nm light with aziPm administration (*n* = 16). Using the 231 µm radius of photoadduction (Fig. 3) that defines a >50% decrease in the measured concentration of adducted ^3^H-aziPm, we estimated a biologically relevant zone of aziPm photoadduction using a sphere with a conservatively larger radius of 250 µm, centered on the UV light source emanating from the localized cannula tips for all subjects (Fig. 4*A*). This plot revealed that 5 of the 6 mice with delayed recovery had photoadduction spheres that bilaterally encompassed the lateral border of the dorsal LC and the parabrachial nucleus (Fig. 4*A*, blue circles), referred to hereafter as the parabrachial-coerulean complex. While this region is by no means the only potential site of anesthetic action in the pons, given the strong correlation between delayed recovery and bilateral photoadduction of the coreulean-parabrachial interface, we chose to perform all subsequent analyses comparing subjects with bilateral cannula within 250 µm of the region (*n* = 5) with subjects where one or more cannula was >250 µm away from the parabrachial-coerulean complex (*n* = 11).

Consistent with prolonged behavioral hypnosis, as measured by tape removal test (Fig. 4*B*), mice receiving bilateral UV light centered within 250 µm of the parabrachial-coerulean complex showed significantly slower median recovery time of 323.68 min (64.21-449.50) compared with subjects in whom at least one of the fiber-optic UV light sources was >250 µm away from the parabrachial-coerulean complex (off-target photoadduction) 15.33 min (3.00-23.87) (Kruskal-Wallis statistic H(2) = 11.29, *p* = 0.0035, Dunn's multiple comparison, parabrachial complex vs distant photoadduction, *p* = 0.0083). In contrast, recovery times for mice with off-target photoadduction of aziPm were indistinguishable from those receiving aziPm without UV photoillumination (Dunn's multiple comparison, distant photoadduction vs no laser, *p* > 0.9999). Thus, localized photoadduction of aziPm encompassing the bilateral parabrachial-coerulean complex profoundly extends the desired anesthetic effect of hypnosis >20-fold.

### Photoadduction of aziPm bilaterally at the parabrachial-coerulean complex prolongs EEG markers of hypnosis without differentially affecting other physiologic effects of hypnotic drugs

**Figure 5.**
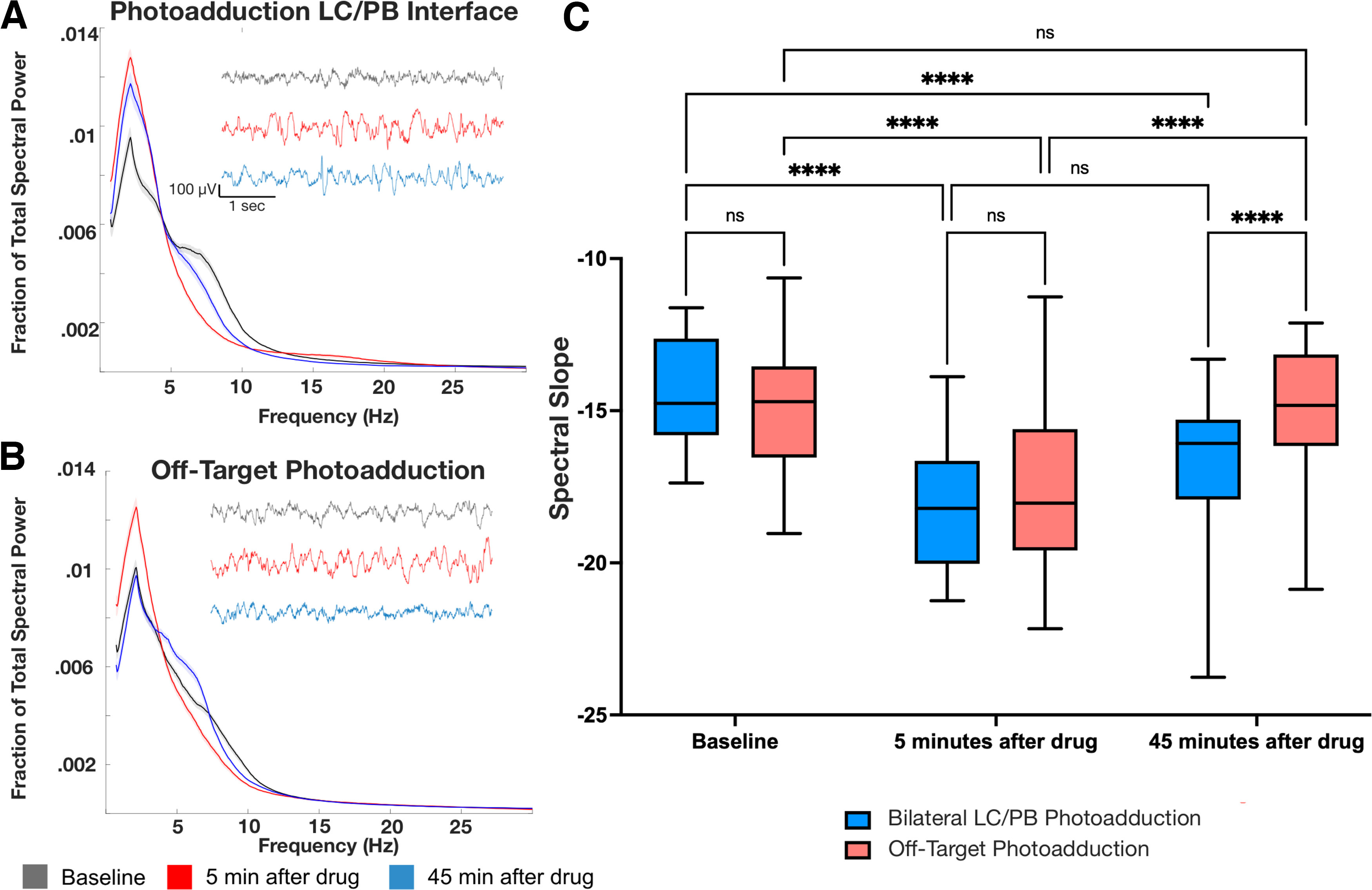
Photoadduction of aziPm in the parabrachial-coerulean complex elicits prolonged EEG signatures of hypnosis compared with off-target photoadduction. ***A***, Mean frontal EEG power spectrum normalized to fraction of total power is shown for parabrachial-coerulean complex photoadduction (*n* = 5) or (***B***) off-target photoadduction (*n* = 8). Insets, Representative 5 s raw EEG traces. Mean and 95% CIs are shown for baseline signal before drug administration (gray), 5 min after beginning drug infusion (red), and 45 min after beginning drug infusion (blue). ***C***, Spectral slope remains decreased in mice with photoadduction encompassing the parabrachial coerulean complex (LC/PB) 45 min after aziPm infusion. ****p < 0.0001.

To determine whether delayed removal of the snout sticker truly reflected an extended state of hypnosis, as opposed to merely reflecting a confound, such as reduced movement, we analyzed murine EEG and EMG. Mice with photoadduction bilaterally targeting the parabrachial-coerulean complex and those with off-target photoadduction both show similar initial slowing of the EEG oscillations. This is consistent with hypnosis following systemic aziPm drug administration (Fig. 5*A*,*B*). However, differences in the location of aziPm photoadduction become apparent over time (two-way repeated-measures ANOVA time × photoadduction location, *F*_(2381)_ = 9.093, *p* = 0.0001). While there is no difference in spectral slope 5 min after administration of aziPm between off-target and parabrachial coerulean complex photoillumination (Sidak's multiple comparisons, *p* = 0.8792), 45 min after photoadduction, the mice receiving off-target UV light exhibit an EEG spectral slope statistically indistinguishable from their baseline (Sidak's multiple comparisons, *p* > 0.9999) and consequently consistent with recovery. Meanwhile, mice that received parabrachial-coerulean complex photoillumination exhibit prolonged changes in EEG spectral slope, consistent with ongoing sedation, that still have not recovered at the 45 min mark (Sidak's multiple comparisons, *p* < 0.0001) (Fig. 5). Hence, EEG correlates of hypnosis are sustained with localized aziPm photoadduction at the parabrachial-coerulean complex.

**Figure 6.**
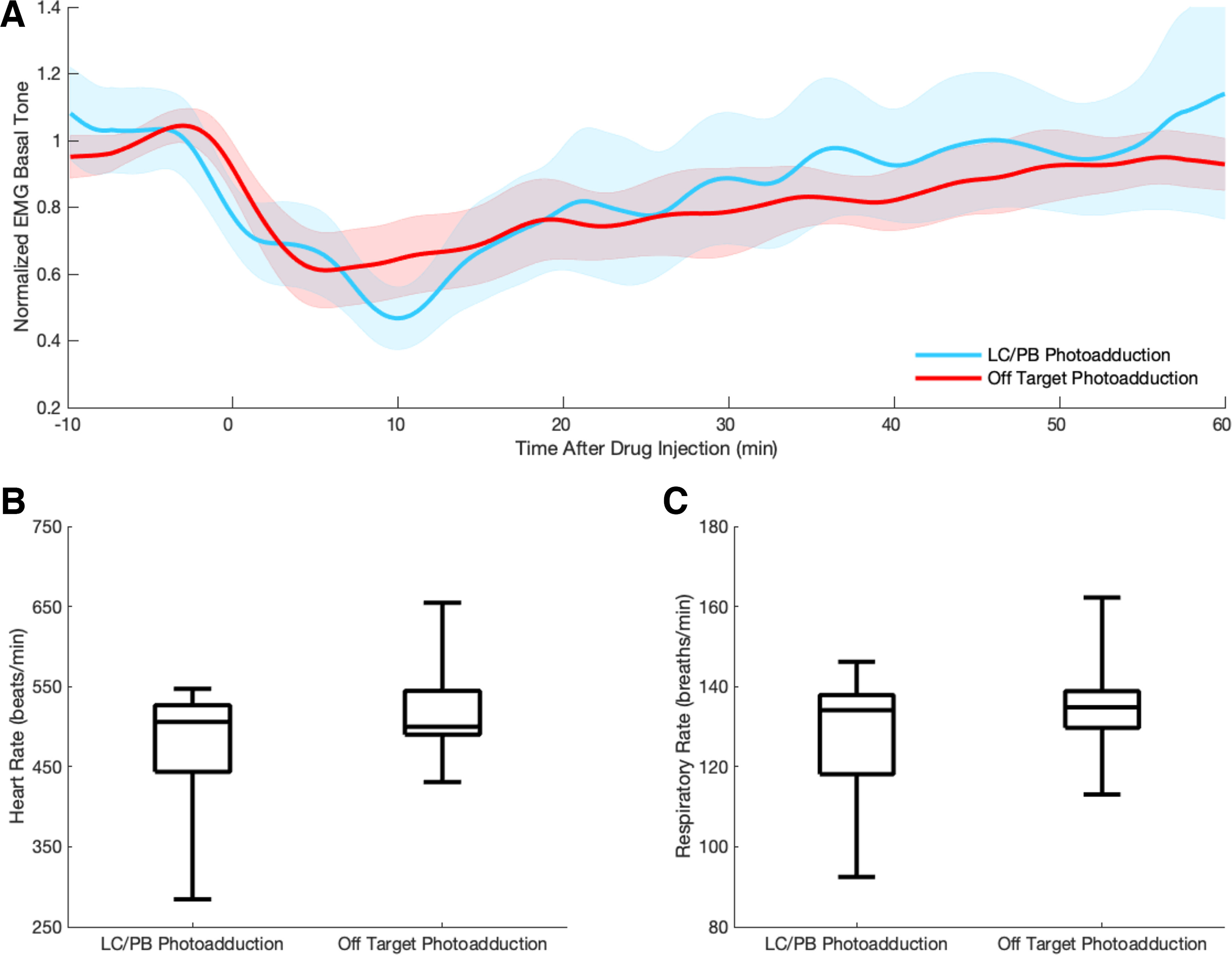
Photoadduction of aziPm in the parabrachial-coerulean complex does not differentially alter EMG tone, heart rate, or respiratory rate compared with off-target photoadduction. ***A***, Mean EMG tone is not significantly different when comparing rostral pons parabrachial-coerulean photoadduction (*n* = 5) and off-target photoadduction (*n* = 8). Standard error of the EMG is shown in the corresponding shaded color. ***B***, Heart rates do not differ over the first 10 min following aziPm delivery between photoadduction groups. ***C***, Respiratory rates do not differ over the first 10 min following aziPm delivery between photoadduction groups.

EMG determined nuchal muscle tone was indistinguishable between mice with photoadduction at the parabrachial-coerulean complex and off-target photoadduction groups (repeated-measures two-way ANOVA, *F*_(419,4609)_ = 0.4991, *p* > 0.9999), suggesting that the prolongation of drug effect seen in behavior and EEG was specific to hypnosis and was not because of motor atonia (Fig. 6*A*). Moreover, photoadduction at the parabrachial-coerulean complex did not produce a differential effect on heart (unpaired two-tailed *t* test, *t* = 0.9004, *p* = 0.3914) or respiratory rates (unpaired two-tailed *t* test, *t* = 0.7905, *p* = 0.4496), arguing against differential kinetics deriving from differing physiologic drug response (Fig. 6*B*,*C*). In 1 of 19 mice that received photoadduction, when the 375 nm illumination was turned on, the mouse immediately ceased respiration and did not recover. Because of the rapid and unexpected death, we were unable to localize the implanted cannula and it was excluded from the analyses.

### Inhibition of noradrenergic cells in the LC by aziPm is potentiated and extended with photoadduction

**Figure 7.**
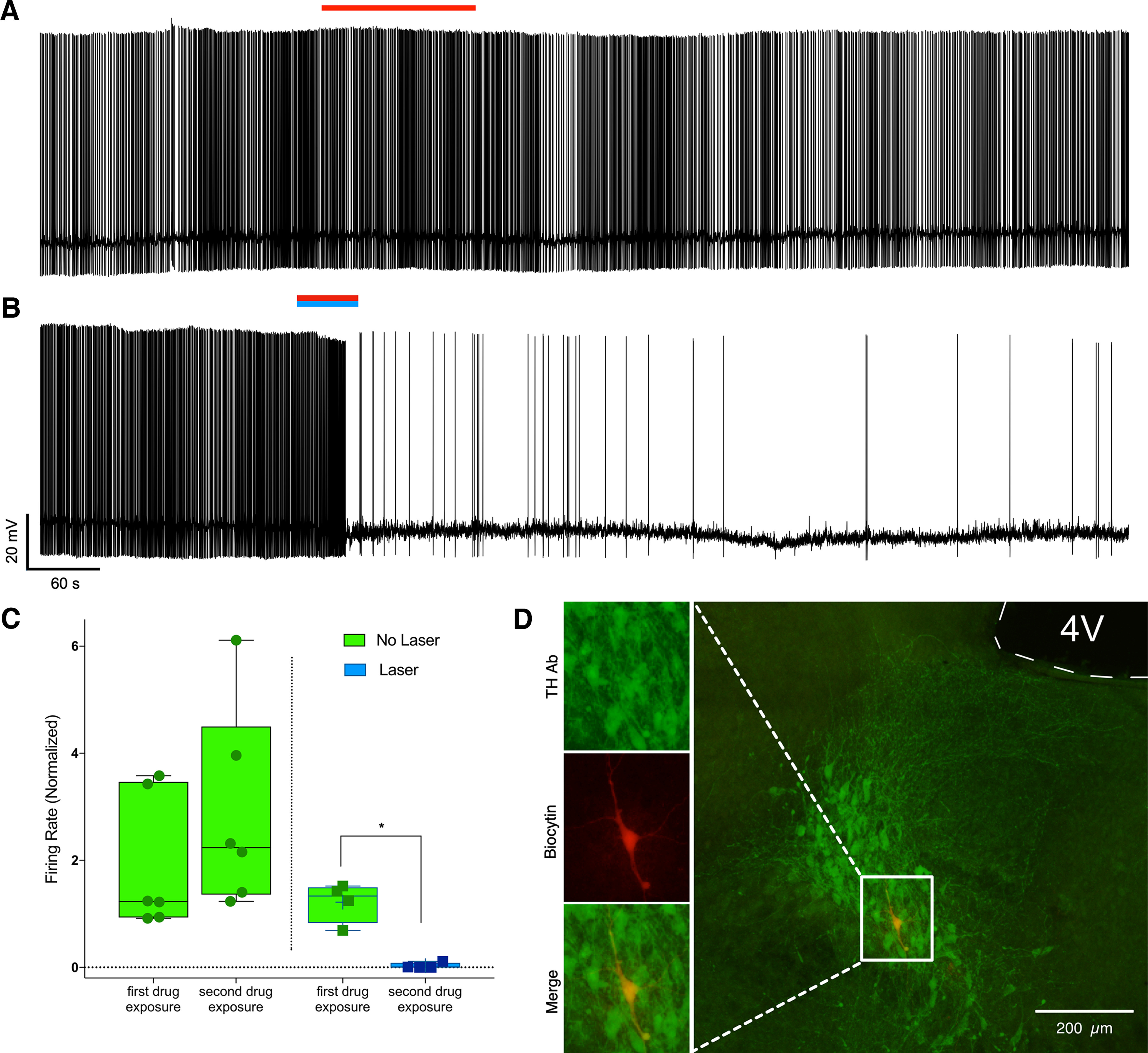
Photoadduction of aziPm in the LC suppresses spontaneous neuronal activity. ***A***, Representative trace of intracellular recording showing minimal effect of 3 min bath application of 10 μm aziPm (red bar) on spontaneous neuronal activity. ***B***, Photoadduction with simultaneous 10 μm aziPm and 375 nm light (blue bar) causes profound suppression of spontaneous firing. ***C***, Suppression of spontaneous activity is not an artifact of multiple exposures, as second aziPm exposures without 375 nm light show no decrease, but rather an increase, in spontaneous firing rates. ***D***, Recorded neurons were confirmed as noradrenergic by immunohistochemical colocalization of biocytin, which marks the recorded neuron (red) and TH (green). *p < 0.05.

We used intracellular current-clamp recordings of pontine brain stem slices to examine the neuron-level effects of *in vitro* photoadduction of aziPm. We focused on AP spiking of LC neurons whose electrophysiological properties are well described (Williams et al., 1988; Chen et al., 1999; Carter et al., 2010; X. Zhang et al., 2010). A 1 min bath application of 10 μm aziPm in aCSF had little effect on spontaneous activity of adrenergic neurons of the LC in the absence of illumination (Fig. 7*A*,*C*) with baseline-normalized mean firing rates of 1.887 and 1.219 after first aziPm washout. However, with 10 uM aziPm bath application together with 375 nm UV photoillumination, noradrenergic LC neurons irreversibly slowed their firing to a baseline-normalized mean firing rate of 0.0293 (two-way ANOVA, *F*_(1,8)_ = 18.40, *p* = 0.0027, Sidak's multiple comparisons test difference for drug and laser first exposure to second exposure *p* = 0.0317). These neurons became virtually quiescent for the duration of the recordings, which lasted up to 90 min following drug washout (Fig. 7*B*,*C*). This was a difference from the non–UV-illuminated neurons, which had a baseline-normalized mean firing rate of 2.863 during the same period, actually slightly increasing after the second versus first aziPm exposure (two-way ANOVA, *F*_(1,8)_ = 18.40, *p* = 0.0027, Sidak's multiple comparisons test difference for drug and laser first exposure to second exposure *p* = 0.0311, Fig. 7*C*). Recorded cells were confirmed as adrenergic *post hoc* by colocalizing TH and biocytin immunohistochemically (Fig. 7*D*).

## Discussion

We demonstrate that regionally confining a lipophilic, low-potency general anesthetic at its *in vivo* site of action in a spatially restricted volume of the mouse brain is sufficient to prolong the state of hypnosis 20-fold. The remarkable extension of anesthesia ensues when photoadduction of aziPm occurs within 250 µm of parabrachial-coerulean complex and arises without accompanying adverse effects, such as respiratory depression, that would otherwise develop with larger systemic drug levels. This extended “optoanesthesia” state is not behaviorally confounded by motor atonia but contains prototypic signatures of hypnosis, including EEG slowing with increased δ and reduced theta power. Protracted recovery following *in vivo* aziPm photoadduction encompassing the parabrachial-coerulean complex suggests a role for this region either in maintaining hypnosis or preventing emergence. Mirroring the potentiation of behavioral hypnosis, localized photoadduction of aziPm in pontine slices proved capable of irreversibly silencing spontaneous APs long after washout of nonphotoadducted drug.

Photoadduction does not discriminate among neuronal subtypes. Hence, the proximate effect of bilateral targeting of parabrachial-coerulean complex is unlikely to be mediated solely by inhibition of adrenergic cerulean neurons, which we confirmed by slice electrophysiological recordings, as opposed to effects on other populations, such as glutamatergic neurons of the parabrachial nucleus (Luo et al., 2018). GABAergic neurons in and around the LC are known to project onto adrenergic LC neurons and are themselves involved in arousal tuning (Breton-Provencher and Sur, 2019). We did not measure the electrophysiological effects of aziPm on parabrachial neurons. However, based on the ability of other anesthetics to depress parabrachial firing by potentiating postsynaptic GABA_A_ currents (Xu et al., 2020), we would expect transient inhibition of the brief bath application of aziPm that also becomes irreversible with UV photoadduction. Thus, as occurs in both rodents and humans with lesions at the parabrachial-coerulean complex (Fischer et al., 2016), potentiation of hypnosis could thus be a combinatorial effect on the GABAergic, glutamatergic, and noradrenergic plus other populations found in the LC and parabrachial nuclei (Fuller et al., 2011; Vazey and Aston-Jones, 2014; Muindi et al., 2016; Qiu et al., 2016; Luo et al., 2018; Wang et al., 2019; Ao et al., 2021).

Additional nearby pontine nuclei, such as the laterodorsal and posterodorsal tegmental nuclei, have known arousal-modulating properties (Scammell et al., 2017). It is possible that targeting these nuclei with photoadducting aziPm could also modulate an anesthetic state. However, based on our modeling of radiolabeled ^3^H-aziPm, we do not predict these nuclei to be encompassed in this study. Of note, the only photoadducted mouse with delayed recovery (64 min) that did not bilateral hit the parabrachial-coerulean complex instead had bilateral targeting of a more ventral region of the LC. As the photoadduction sphere in this animal missed the parabrachial-coerulean complex, it was included with the “off-target” cohort (Fig. 4).

One potential weakness of our modeling was the presupposition that the photoadduction volume was perfectly spherical (Figs. 3, 4). The actual adducted volume likely differs, which would affect our predicted borders. Nevertheless, *in vitro* and *in vivo* ^3^H-aziPm studies confirm that irreversible binding of ^3^H-aziPm rapidly decreases as a function of distance from the epicenter of the UV light source. UV light power decreased to 50% 203 µm from the source. Moreover, with distances exceeding a 231 µm radius, we estimate that residual photoadducted concentration would fall below the pharmacologically relevant concentration of 0.55 μm azi-propofol based on the subsequent logic and literature. The maximum concentration of irreversible ^3^H-aziPm binding immediately adjacent to the fiber tip was 11.3 nm (Fig. 3). The radiolabeled ^3^H-aziPm dose was ∼1% of aziPm used in behavioral experiments. All other exposure parameters were identical between behavioral experiments with ^3^H-aziPm and aziPm. Given that the limiting factor in photoadduction is optical power and that a consistent fraction of total available photoligand should undergo adduction at a given power level, the upper range of adducted aziPm concentrations in the behavioral experiments is predicted to be 100 times larger: 1.13 μm. As aziPm has roughly half the molar potency of propofol (Fig. 1), our aziPm concentrations are comparable to corresponding hypnotically relevant propofol dose (Smith et al., 1994; Harris et al., 2006). Our cutoff for minimum relevant concentration (a 50% decrement of adduction occurs at 231 µm from the epicenter and corresponds to 5.51 nm of ^3^H-aziPm) is similarly based on physiologically relevant drug levels. This adducted concentration of radiolabeled ^3^H-aziPm given at 1 mg/kg is equivalent to 0.55 μm of aziPm adducted following the 100 mg/kg actual IV dosing. This minimum cutoff conservatively compares to the minimum concentration in slice recordings where 0.63 μm propofol has a neurophysiologic effect (Gredell et al., 2004). Hence, our 250 µm radius yields a conservatively large estimate of the extent of relevant photoadduction.

Using *in vivo* photoaffinity ligands to gain mechanistic insight about relevant neuronal circuitry provides advantages over existing techniques, including lesion studies, microinjection, reverse microdialysis, and optogenetics. Lesion studies may result in nonspecific anatomic destruction, may induce potential compensatory changes in circuit structure and activity, and may not specifically target the cells affected by the drug. While combining genetic and anatomic targeting of lesions increases specificity and potential relevancy, compensatory plasticity remains a confounder and any differential drug effects on separate neuronal subtypes within a circuit will be masked by the blunt effect of a lesion. Photoaffinity ligands are designed to interact with the targets of the parent drug, closely mirroring the effects seen with systemic administration of drug (Woll et al., 2016). Rapid drug action with these ligands precludes longer-term plastic changes that emerge with lesions. Intracerebral microinjection produces a drug gradient that varies over space and time in less predictable ways. Reverse microdialysis can mitigate the concentration change immediately surrounding the dialysis probe, but regions beyond the probe's immediate neighborhood remain exposed to a not-easily controlled drug gradient. While *in vivo* photolabeling also creates a strong gradient of bound drug (Fig. 3), the comparatively very steep decrement in drug concentration is determined by light penetration and, following photoadduction, is fixed. Although optogenetics offers tight temporal control and targeting specificity (Häusser, 2014), it requires prior knowledge of the relevant neural population and is ill suited to simultaneous targeting the multiple populations that could be concurrently affected by a drug. Photoaffinity ligands largely retain the ability to affect the same neuronal subtypes as their parent compounds, permitting the same complex modification of neural circuits as the CNS drugs from which they are derived.

In addition to being a tool for identifying brain regions involved in the therapeutic action of drugs, there is anecdotal evidence of photoaffinity ligands being able to prolong or enhance some off-target drug effects as well. Photo-activatable compounds are certainly able to do the inverse, avoiding adverse systemic drug effects with local activation (López-Cano et al., 2021). In the case of aziPm's parent compound, propofol, respiratory depression is a well-known adverse effect. The overwhelming majority of mice that received *in vivo* photoadduction did not manifest any overt respiratory depression following the 20 mg/kg/min IV infusion of aziPm given over 5 min (Fig. 6*C*). One limitation of our study was our inability to localize implanted cannulas in 2 of 19 photoadducted mice, including 1 mouse that died unexpectedly because of apnea temporally correlated with UV photoadduction. Given that the Kölliker-Fuse nucleus is both capable of provoking apnea in anesthetized rodents and is lateral to the parabrachial nucleus (Song et al., 2010), we presume that photoadduction may have caused irreversible dysfunction in this nucleus capable of modulating respiratory drive. Another limitation of our study is the relatively small number of 5 mice with photoadduction encompassing neurons of the parabrachial-coerulean complex. Our study cannot exclude the possibility that additional pontine nuclei would could not similarly or more dramatically prolong the duration of an anesthetic state.

Markedly prolonged recovery following aziPm photoadduction of the parabrachial-coerulean complex paralleled that previously described with whole-organism aziPm photoadduction in tadpoles (Weiser et al., 2013). In both cases where ligand is covalently adducted, the relevant question is why do drug effects terminate at all? One possible explanation is that drug effect in these cases is only terminated with receptor internalization. One known target of the alkylphenols, the GABA_A_ receptor, exhibits increased endocytosis in the presence of agonists (Chaumont et al., 2013); therefore, photoadducted receptors would be more likely to be internalized and digested. Radiolabeled ^3^H-aziPm could be used in cell culture to investigate the role of endocytosis in terminating the drug effect.

While we chose to photoadduct in the rostral pons as proof of principle in studying systemic effects of local CNS anesthetic photolabel adduction, this same approach could be extended to multiple other brain regions previously shown to be involved in arousal and as targets for anesthetic effects. Numerous and disparately located CNS centers, including other areas of the pons, medulla, midbrain, hypothalamus, basal forebrain, thalamus, and cortex, have been demonstrated to be critical for anesthetic action or emergence from anesthesia (Devor and Zalkind, 2001; Abulafia et al., 2009; Luo and Leung, 2009; Moore et al., 2012; Solt et al., 2014; Z. Zhang et al., 2015; Gelegen et al., 2018; Pal et al., 2018; Gao et al., 2019; Wang et al., 2019; J. Y. Li et al., 2022). Our results confirm that *in vivo* photoadduction produces local therapeutic-level concentrations of drug in ROIs. Therefore, an optoanesthesia approach could prove a potent new tool in the neuroanatomic investigation of anesthetic action. We demonstrate the potential for *in vivo* use of photoaffinity ligands to investigate multiscale CNS drug effects. However, the current requirement for an implanted fiber for photoadduction limits immediate clinical utility. Borrowing therapeutic ideas from oncology, a combinatorial approach using near-infrared light able to penetrate into the CNS as well as upconverting nanoparticles (He et al., 2015; Yu et al., 2016), one may imagine a noninvasive route to deploying photoactive anesthetic compounds in the clinical realm in the future.
